# Transcriptomic mortality signature defines high-risk neonatal sepsis endotype

**DOI:** 10.3389/fimmu.2025.1601316

**Published:** 2025-06-27

**Authors:** Faris N. Al Gharaibeh, Min Huang, James L. Wynn, Rishikesan Kamaleswaran, Mihir R. Atreya

**Affiliations:** ^1^ Division of Neonatology, Cincinnati Children’s Hospital Medical Center, Cincinnati, OH, United States; ^2^ Department of Pediatrics, University of Cincinnati College of Medicine, Cincinnati, OH, United States; ^3^ Department of Biomedical Informatics, Emory University School of Medicine, Atlanta, GA, United States; ^4^ Division of Neonatology, Department of Pediatrics, University of Florida, Gainesville, FL, United States; ^5^ Department of Biomedical Engineering and Electrical & Computer Engineering, Duke University, Durham, NC, United States; ^6^ Division of Critical Care, Cincinnati Children’s Hospital Medical Center, Cincinnati, OH, United States

**Keywords:** neonatal sepsis, mortality, gene-expression profiling, endotypes, neutrophils

## Abstract

**Introduction:**

Neonatal sepsis remains a leading cause of global childhood mortality, yet treatment options are limited. Clinical and biological heterogeneity hinders the development of targeted therapies. Gene-expression profiling offers a potential strategy to identify neonatal sepsis subtypes and guide targeted intervention.

**Methods:**

We performed secondary analyses of publicly available gene-expression datasets. Differential gene expression analysis and T-distributed Stochastic Neighbor Embedding (t-SNE) identified biologically relevant patient clusters. Mortality and organ dysfunction were compared across clusters to determine clinical relevance.

**Results:**

We identified three endotypes of neonatal sepsis based on the 100 gene expression mortality signature, distinguishing five non-survivors from 72 survivors across datasets. Compared with other endotypes, Endotype A was associated with high mortality (22% vs. 0%, p=0.003) and cardiac dysfunction (61% vs. 31%, p=0.025). Pathobiology among endotype A patients was primarily driven by neutrophil progenitors.

**Conclusions:**

Gene-expression profiling can be used to disentangle neonatal sepsis heterogeneity. Dysregulated hyperinflammatory response with emergency granulopoiesis was pathognomonic of high-risk endotype A. Pending further validation, gene-expression-based subclassification may be used to identify at-risk neonates and inform the selection of targeted sepsis therapies.

## 1 Introduction

Neonatal sepsis remains a major cause of morbidity and mortality worldwide. Infections account for a significant proportion of neonatal deaths, with pooled estimates indicating a high mortality rate of 10–29% (1). In the U.S., one in four extremely preterm neonates experience at least one episode of pathogen-confirmed sepsis during their Neonatal Intensive Care Unit (NICU) stay (2). Beyond the acute phase, survivors face long-term complications that extend well beyond the NICU, contributing to adverse neurodevelopmental outcomes and prolonged healthcare needs (3). Despite its substantial impact, the host response to sepsis and its relationship to neonatal sepsis outcomes remain poorly understood. Furthermore, biological heterogeneity among patients with neonatal sepsis has limited the development of targeted treatments.

Gene-expression profiling has successfully identified reproducible disease subtypes among critically ill adults and pediatric populations. *Wong et al.* first detailed pediatric septic shock endotypes with prognostic and therapeutic relevance (4). The high-risk endotype A has an increased risk of mortality and detrimental response to adjunct corticosteroids (5). Similar studies have since been replicated among adult populations (6–8). While gene-expression profiling has been conducted in neonates, demonstrating age-related development differences in host responses to sepsis (9, 10). To the best of our knowledge, no prior studies have attempted to identify neonatal sepsis subtypes.

In this study, we leveraged publicly available gene-expression datasets of neonatal sepsis to test the hypothesis that sepsis-related mortality defines a unique gene signature, providing insight into disease heterogeneity.

## 2 Methods

### 2.1 Gene expression datasets

We conducted a secondary analysis of publicly available whole-blood gene expression datasets and de-identified data from neonates with sepsis. Accordingly, the study was exempt from approval by the Institutional Review Board (IRB). Two authors (FA and MA) identified Gene-Expression Omnibus datasets available in the National Library of Medicine Geo Dataset repository, using the following MeSH terms: Sepsis and Neonate, and excluding species other than Homo Sapiens. The search yielded thirteen datasets. Eleven were excluded: Five included only certain cell types, two because they did not include neonates with sepsis, and four because they included patients of older age groups admitted to a setting other than the NICU. The two datasets included in the analysis were those published by *Smith et al.* (GSE25504) (11) and *Wynn et al.* (GSE69686) (12). The former conducted two separate batches of gene-expression data. Combat co-normalization using controls (COCONUT) (13) was applied to selected gene-expression data to eliminate the batch effect.

### 2.2 Primary and secondary outcomes definitions

Clinical definitions were made based on the available de-identified metadata from the datasets. Our primary outcome was mortality. Since we did not have access to the reported cause of death and to ensure that we do not include late deaths that occurred due to co-morbidities other than sepsis, we chose 10-day mortality. Secondary outcomes include cardiac, respiratory, and hematologic dysfunctions and identification of a causative pathogen on blood culture. We defined organ dysfunctions as follows: Cardiac dysfunction: Reduced perfusion and hypotension requiring fluid resuscitation or vasoactive support; Respiratory dysfunction: Supplemental oxygen or intubation or mechanical ventilation; Hematologic dysfunction: Platelet count < 150 X 10^3^/mm^3^.

### 2.3 Differential gene-expression signature based on neonatal sepsis mortality

Limma package (v.3.54.2) in R was applied to identify differentially expressed genes (DEGs) according to the primary outcome. A predetermined log2 fold change > ± 1 and a Benjamin Hochberg adjusted false discovery rate (FDR) ≤ 0.05 was used. Heatmap and volcano plots were used to visualize DEGs. We determined enriched biological processes using REACTOME pathway analyses.

### 2.4 Identification of upstream regulators of neonatal sepsis mortality

To identify key transcriptional regulators among high-risk patients, we submitted the gene list associated with neonatal sepsis mortality to the Chip Enrichment Analysis (ChEA3) portal (https://maayanlab.cloud/chea3/) to predict transcription factors (TFs) anticipated to regulate gene expression (14). The most notable TF is denoted by the lowest mean rank, which indicates the TF predicted by ChEA3 to interact most with the submitted gene lists after searching across multiple libraries, including ENCODE, GTEx, ARCHS4, and ReMap. Additionally, we submitted the gene list associated with neonatal sepsis mortality for Ingenuity Pathway Analysis (15) (QIAGEN Inc., https://digitalinsights.qiagen.com/IPA) to identify upstream regulators and mechanistic networks that could influence gene-expression patterns, focusing on direct interactions between regulators and selecting TFs with highest activation z-scores and p<0.001.

### 2.5 Identification of neonatal sepsis endotypes based on mortality signature

T-distributed Stochastic Neighbor Embedding (t-SNE) was used to project gene-expression data and identify neonatal sepsis endotypes based on the mortality gene-expression signature. To determine the optimal number of clusters, we used the Silhouette Score to determine the overall goodness-of-fit of the cluster structure.

### 2.6 Testing clinical relevance of neonatal sepsis endotypes

Demographic and outcome measures were measured across endotypes to determine their clinical relevance. Categorical variables are described as total numbers and percentages and compared using Fisher’s exact or Chi-square test. Non-parametric continuous variables were described using the median and interquartile range and compared using the Mann-Whitney or Kruskal-Wallis test as appropriate. Given the small and imbalanced nature of the dataset with perfect class separation of mortality across endotypes, we adjusted for baseline characteristics, including gestational age and sex, relevant to the primary outcome using Firth’s logistic regression. Analyses were conducted using GraphPad Prism v10.4 (GraphPad Software, Boston, Massachusetts USA, www.graphpad.com) and RStudio v.1.4 (RStudio Team, Massachusetts USA).

### 2.7 Inferring differences in cell type abundance and signaling associated with endotypes

To assess differences in cell type abundance between endotypes, we utilized CIBERSORTx (16), applying the gene-expression signature linked to neonatal sepsis mortality. Recognizing that cellular abundance and signaling may be discordant, we addressed this limitation by integrating data from a single-cell RNA sequencing (scRNAseq) study of septic adults conducted by *Kwok et al. (* 17) To quantify gene expression changes, we employed a previously established method to compute a composite gene score (18), defined as the geometric mean of the 96 overexpressed genes minus the geometric mean of the four repressed genes. These genes were identified through differential expression analysis and were also present in the scRNAseq dataset. We then projected this scaled composite score onto the visual projection of the scRNAseq dataset to infer the cell types driving biological differences between neonatal sepsis non-survivors and survivors.

### 2.8 Identification of genes distinguishing endotypes

The most differentially expressed genes distinguishing endotype A from other endotypes were identified based on the lowest FDR-adjusted p-values as candidate biomarkers for future validation.

### 2.9 Ethics statement

This study does not involve human or animal subjects. Therefore, consenting or ethical approval is not indicated.

## 3 Results

### 3.1 Demographics of patients included

Seventy-seven neonates were included in the analysis, of whom five (6%) died from sepsis. There were no differences in gestational age at birth, sex, onset of infection (early vs. late), pathogen type, and total white blood cell count (WBC) when comparing non-survivors and survivors, as shown in **Table 1**.

**Table 1:** Clinical variables comparing non-survivors and survivors in the study.

Characteristic	Non-survivors n=5 (6%)	Survivors n=72 (94%)	p-value
GA at Birth Weeks [IQR]	27 [26-29]	28 [27-31]	0.21^a^
Sex at Birth: Female male	1 (20%) 4 (80%)	42 (58%) 30 (42%)	0.64^b^
Early vs. Late	2 (40%)	22 (31%)	0.64^b^
Pathogen Identified	5 (100%)	52 (72%)	0.32 ^b^
Total White Count/mm^3^ [IQR]	9900 [1700-15000]	10150 [6675-15475]	0.41^a^

a Calculated using the Mann-Whitney test.

b Calculated using the Fisher’s exact test.

### 3.2 Differentially expressed genes distinguish neonatal sepsis non-survivors and survivors

**Figure 1:**
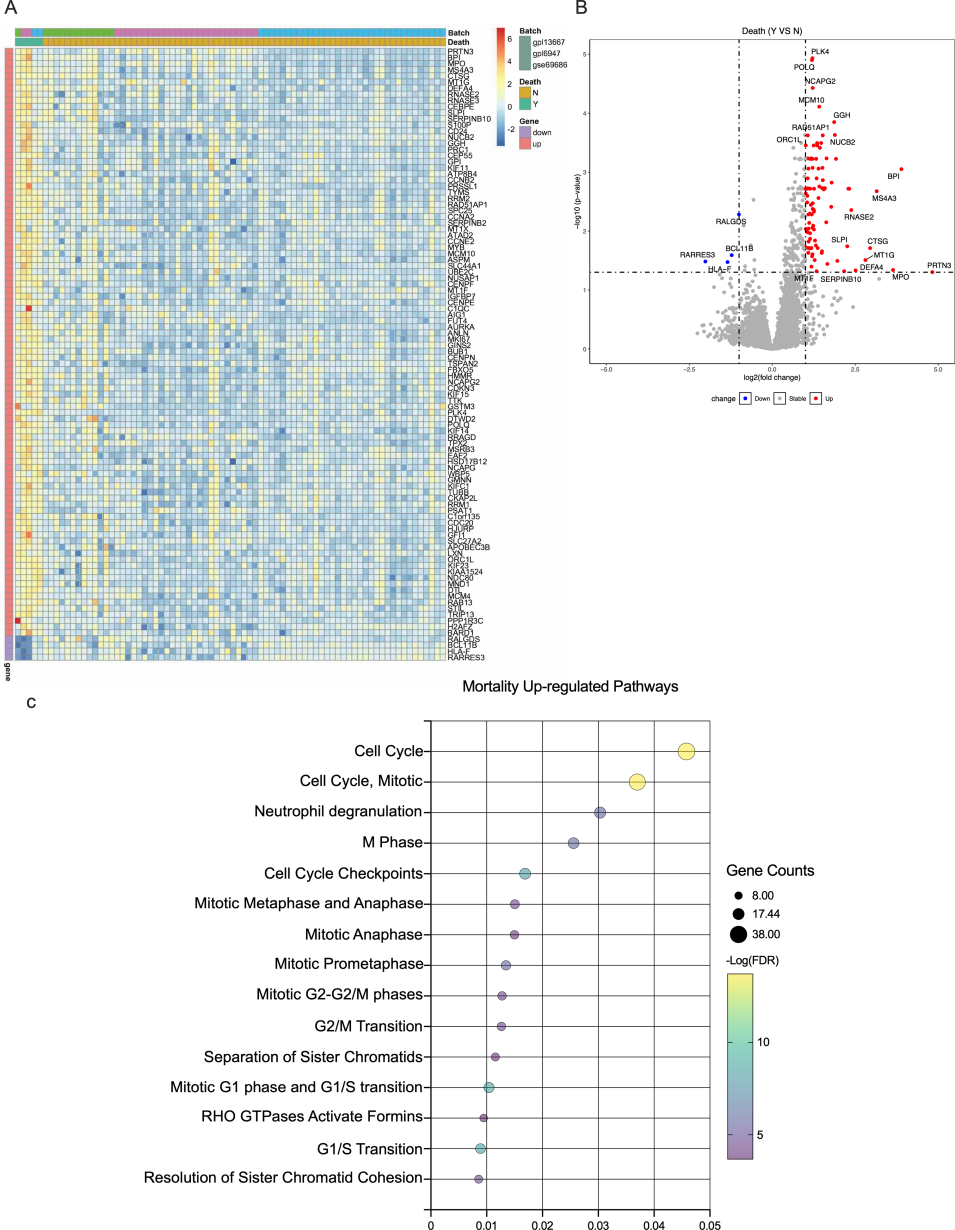
Gene expression according to neonatal sepsis mortality. **(A)**. Heatmap of the mean expression of top differentially expressed genes according to mortality. **(B)**. Volcano plot of differentially expressed genes, the horizontal dotted line represents a -log(p-value) of 1·2, and the vertical ones represent a log (fold change) of ±1. **(C)**. Upregulated REACTOME pathways comparing neonatal sepsis non-survivors and survivors.

We identified 100 differentially expressed genes, of which 96 were overexpressed and four were repressed, distinguishing neonatal sepsis non-survivors from survivors (**Supplementary Table 1**). Upregulated genes among non-survivors were predominantly involved in cell cycle-related processes. **Figures 1A-C** show a heatmap, volcano plot, and upregulated biological pathways.

### 3.3 Transcription factor analyses reveal upstream regulators of neonatal sepsis mortality

We identified 34 TFs inferred to be activated among neonatal sepsis non-survivors. (**Supplementary Table 2**) The top 15 TFs identified were LTF, CEBPE, E2F8, ZNF215, MYB, E2F7, ZNF93, RFX8, CENPA, ZNF695, GFI1, MYBL2, FOXM1, ZNF519, and ZNF124 (**Supplementary Figure 1**). The top 15 TFs identified through IPA analyses were TBX3, E2F4, TCF3, ZBTB17, CCND1, TP53, E2F3, FOXM1, MYOD1, and E2F1 (**Supplementary Table 3**).

### 3.4 Neonatal sepsis endotypes with distinct biological and clinical profiles

**Figure 2:**
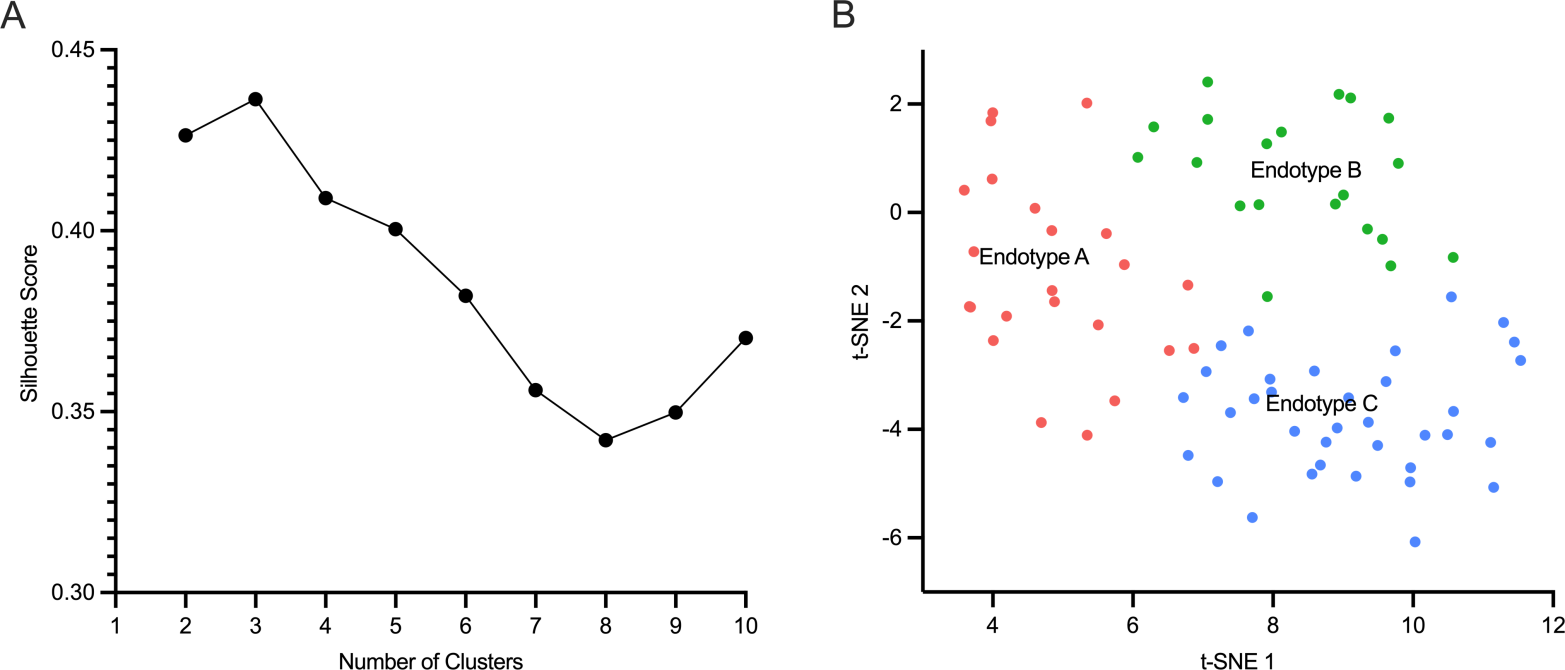
Neonatal sepsis endotypes. **(A)**.The number of optimal clusters (n=3) of neonatal sepsis patients is shown on the x-axis, and the Silhouette score is shown on the y-axis. **(B)**. Neonates were allocated to three clusters or endotypes based on their gene-expression profile in relation to sepsis mortality signature: Endotype A (pink), endotype B (green), and endotype C (blue).

Silhouette Score analysis showed that the ideal number of clusters based on the cohort gene-expression data was three, as shown in **Figure 2A**. The relatedness of each patient’s gene expression profile to the mortality signature was projected using t-SNE and is shown in **Figure 2B**, revealing three distinct clusters. Based on t-SNE coordinates, all patients were subsequently assigned to one of three endotypes – A, B, or C.

Neonates belonging to endotype A had a mortality rate of 5/23 (22%) compared to 0/20 (0%) and 0/34 (0%) for clusters B and C, respectively, p=0.003, as shown in **Table 2**. The adjusted odds of neonatal sepsis mortality in endotype A after adjusting for confounding factors was aOR 24.4 (95% CI 2.6-3253.5) compared to the other endotypes, p 0.003. Neonates classified as endotype A were born earlier at 27 weeks gestation [IQR 25–29 weeks] compared to 28 weeks [IQR 27–30 weeks] and 30 weeks [IQR 27-35] compared to endotypes B and C, respectively, p=0.01. All neonates assigned to endotype B had positive pathogen confirmation 20/20 (100%) compared to 17/23 (74%) and 20/34 (59%) in endotypes A and C, respectively, p=0.004. Although pathogen identification differed between endotypes, the rate of gram-negative pathogens was not statistically different according to the endotype. Neonates assigned to endotype C had more early-onset sepsis 18/34 (53%) compared to 4/23 (17%) and 2/20 (10%) in endotypes A and B, respectively, p=0.001.

**Table 2:** Characteristics according to endotype.

Characteristic	Cluster A (n=23)	Cluster B (n=20)	Cluster C (n=34)	p-value
Mortality	5/23 (22%)	0/20 (0%)	0/34 (0%)	0.003^a^
GA at Birth Weeks [IQR]	27 [25-29]	28 [27-30]	30 [27-35]	0.21^b^
Sex at Birth: Female male	9 (39%) 14 (61%)	10 (50%) 10 (50%)	12 (35%) 22 (65%)	0.56^a^
Early vs. Late	4/23 (17%)	2/20 (10%)	18/34 (53%)	0.001^b^
Pathogen Identified	17/23 (74%)	20/20 (100%)	20/34 (59%)	0.004^a^
Gram-Negative Pathogen	3/17 (18%)	4/20 (33%)	4/20 (33%)	>0.99 ^a^
Cardiac Dysfunction	14/23 (61%)	4/20 (20%)	13/34 (41%)	0.025^a^
Respiratory Dysfunction	18/23 (78%)	13/20 (65%)	20/34 (59%)	0.311^a^
Hematologic Dysfunction	10/10 (50%)	10/20 (50%)	3/8 (38%)	0.257 ^a^
Total White Count/mm^3^ [IQR]	11250 [5950-18475]	13350 [8150-16150]	8500 [5700-12150]	0.07^b^

Neonates in cluster A experienced more death and cardiac dysfunction compared to others.

a Calculated using the Chi-square test.

b Calculated using the Mann-Whitney test.

For secondary outcomes, respiratory and cardiac dysfunction data were available for the entire cohort, and hematologic dysfunction data for 42/77 (55%) neonates. Neonates in endotype A had a higher rate of cardiac dysfunction, 14/23 (61%), compared to 4/20 (20%) and 13/34 (41%) in endotypes B and C, respectively, p=0.03. The rate of respiratory dysfunction and hematologic dysfunction was comparable across endotypes: 18/23 (78%) in endotype A, 13/20 (65%) in endotype B, and 20/34 (59%) in endotype C, p=0.311 and 10/14 (71%) in endotype A, 10/20 (50%) in endotype B, and 3/8 (38%) in endotype C, p=0.26, respectively.

**Figure 3:**
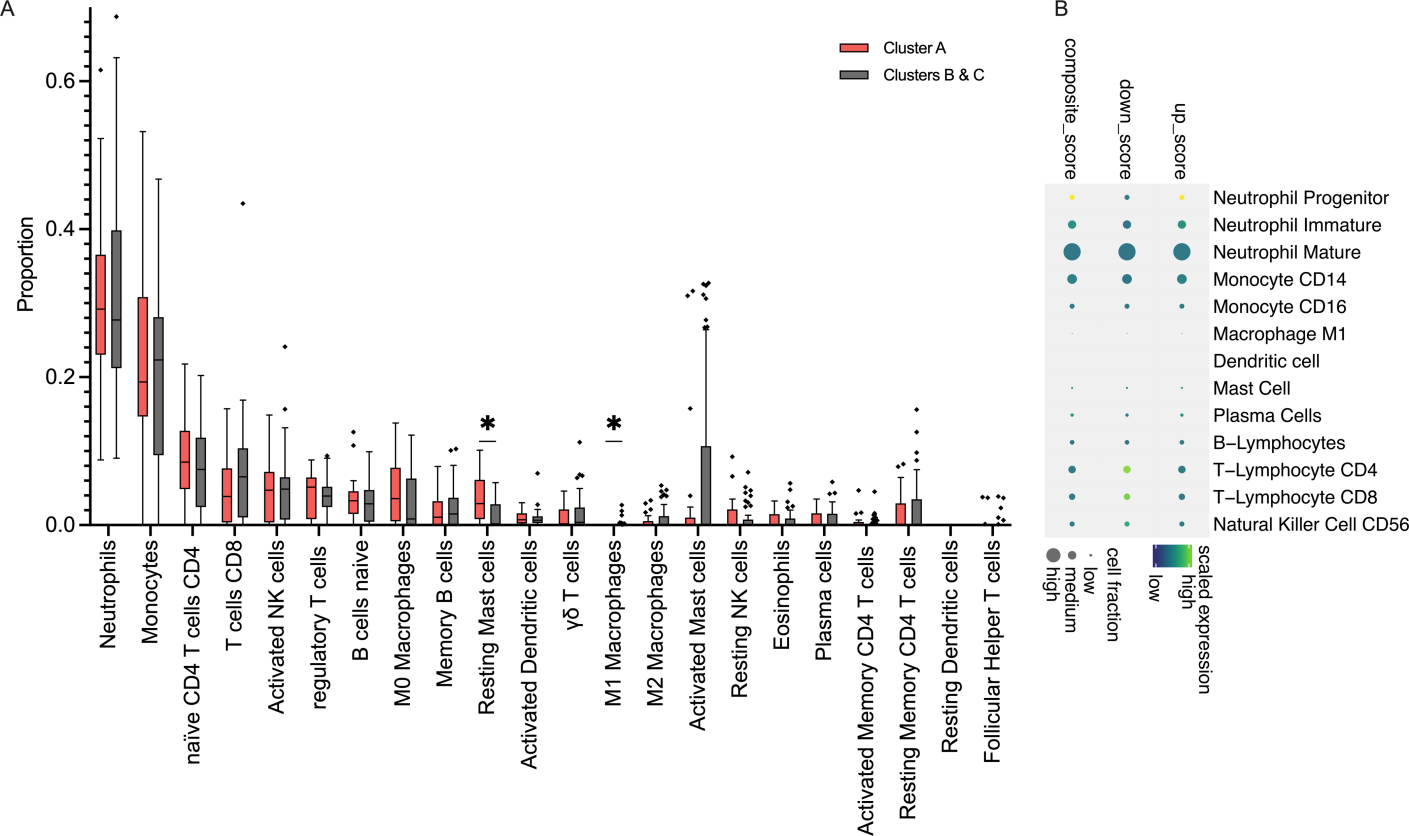
Immune dysregulation of neonatal sepsis endotype A **(A)**. Results of CIBERSORTx showing an inferred abundance of cell types comparing Endotype A vs. others; proportion of subsets are shown as Tukey bars. *: p <0.05. **(B)**. Composite gene expression scores in Endotype A, relative to other endotypes, showing up-regulation of Neutrophil Progenitor signaling and concomitant downregulation of T-Lymphocyte signaling.

**Figure 4:**
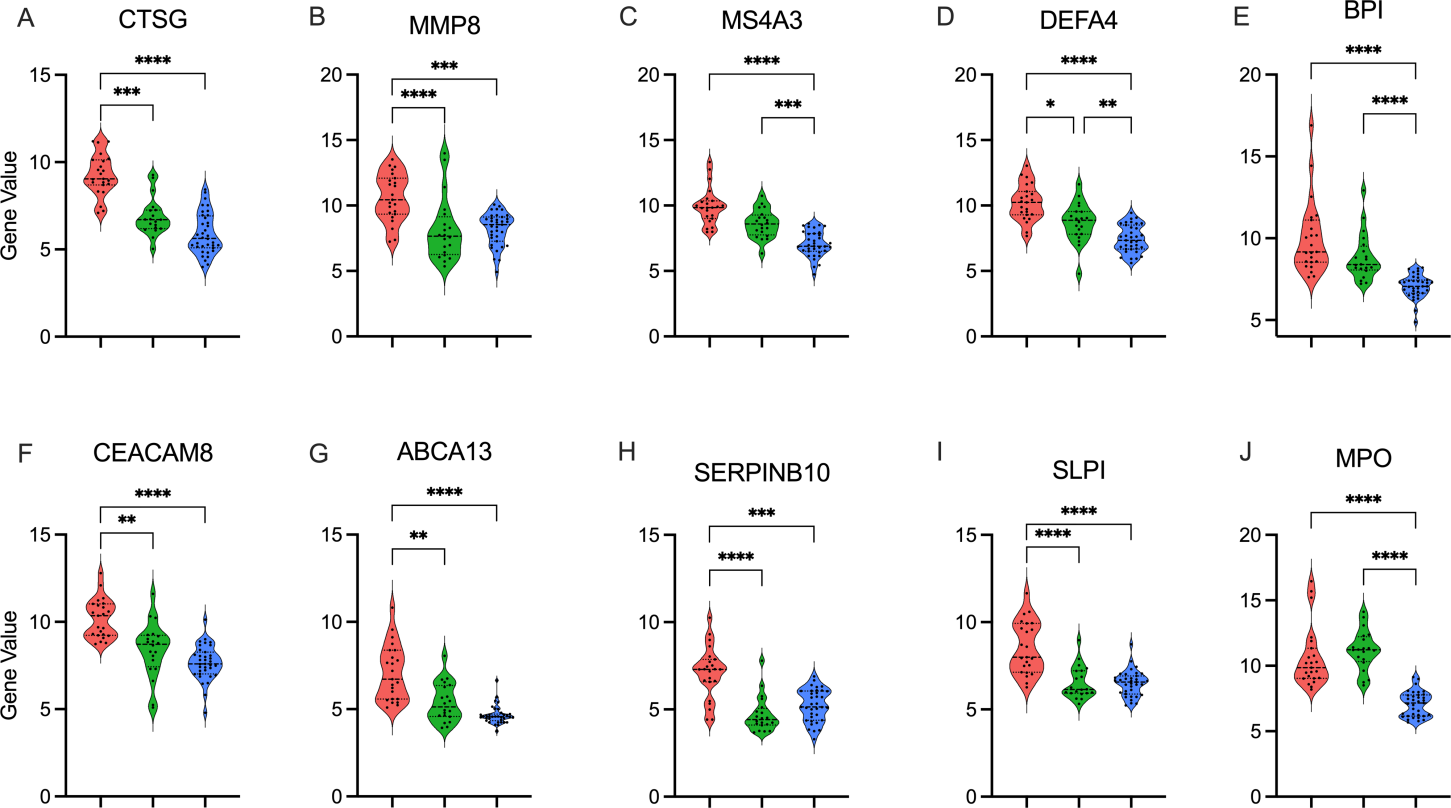
Top gene candidates defining endotype A Top 10 genes distinguishing neonatal sepsis endotypes in descending significance: **(A)**. *CTSG*, **(B)**. *MMP8*, **(C)**. *MS4A3*, **(D)**. *DEFA4*, **(E)**. BPI, **(F)**. CEACAM8, **(G)**. ABCA13, **(H)**. SERPINB10, **(I)**. SLP1, and **(J)**. MPO. Gene expression is shown as violin plots for each cluster A: Endotype A; pink, B: Endotype B; green, C: Endotype C; blue). p value calculated using the Kruskal-Wallis test with Dunn’s correction. *: p < 0.05, **: p <0.01, ***: p <0.001, p<0.0001: ****.

Neonates had no differences in total WBC counts across endotypes. Moreover, CIBERSORTx analyses did not identify significant differences in the major cell types comparing endotypes, including Neutrophils (Neu), Monocytes, B- and T-lymphocytes, or NK cells, comparing detrimental endotype A vs others, as shown in **Figure 3A**. However, inference from the scRNAseq dataset indicated that endotype A was characterized by predominantly Neu progenitor-driven signaling and concomitant downregulation of CD4 and CD8 lymphocyte signaling, as shown in **Figure 3B**. Finally, the top 10 genes distinguishing neonatal sepsis endotypes *CTSG*, *MMP8*, *MS4A3*, *DEFA4*, *BPI*, *CEACAM8*, *ABCA13*, *SERPINB10*, *SLP1*, and *MPO* are shown in **Figures 4A-J**.

Finally, we compared neonates who survived to those who did not in cluster A, and we did not find any differences related to gestational age or sex at birth, timing of sepsis, or respiratory or cardiac dysfunctions (**Supplementary Table 4**).

## 4 Discussion

We analyzed publicly available gene-expression datasets to define a neonatal sepsis mortality signature and identify transcription factors linked to immune dysregulation. Gene-expression profiling revealed distinct neonatal sepsis endotypes, with endotype A associated with significantly higher mortality and cardiac dysfunction. While cell abundance analysis showed no major differences, single-cell RNA sequencing inference indicated that endotype A was driven by neutrophil progenitor signaling and suppressed CD4+ and CD8+ lymphocyte signaling. These findings suggest that functional immune dysregulation, rather than overt differences in cell abundance, contributes to poor outcomes in neonatal sepsis. Our study underscores the potential of gene-expression profiling for disease subtyping and precision medicine in neonatal sepsis.

Previous gene expression studies have primarily focused on differentiating infected from uninfected controls. By aggregating datasets, we identified a distinct gene-expression signature associated with neonatal sepsis mortality. However, mortality was still a rare outcome, likely due to the challenges of studying critically ill neonates, consent limitations, and sampling challenges within the included datasets. We identified key TFs implicated in driving neonatal sepsis mortality with Forkhead box M1 (FOXM1) – a master regulator of cell cycle regulation and T-lymphocyte survival (19) – emerging as the only TF identified through both computational approaches used. Furthermore, animal models of lung injury have shown that FOXM1 is essential in regulating neutrophil response and secretion of MPO and cathepsin G (20), both of which were enriched in neonates that did not survive sepsis. Future mechanistic studies are warranted to elucidate the role of FOXM1 and other TFs in immune dysregulation and explore their potential as therapeutic targets in neonatal sepsis.

We identified three robust clusters of neonatal sepsis, which we labeled as endotypes, given their pathophysiologic distinctiveness. Patients assigned as endotype A had significantly higher adjusted odds of mortality after controlling for gestational age and sex. Notably, neonates belonging to endotype A had lower rates of early-onset sepsis and pathogen-identification rates than those with endotypes B and C. Our data contradict prior reports have indicated higher mortality with early- vs late-onset cases (21), and differences when comparing culture-positive vs -negative sepsis (22).

Our findings revealed an overactive innate immune response driven by neutrophil progenitors and repression of T-lymphocyte signaling in detrimental endotypes, suggesting that immune dysregulation rather than gestational age or pathogen detection is the primary determinant of neonatal sepsis outcomes.

Lastly, we identified genes that reliably distinguished neonatal sepsis endotypes, with the top two being cathepsin G (CTSG) and matrix metalloprotease 8 (MMP8). Cathepsin G (CTSG) is a protease presented on or released from many immune cells, mainly neutrophils, upon activation (23). It plays a significant role in inflammation and platelet aggregation, and higher plasma levels of cathepsin G were linked to more severe illness and death from SARS-Cov-2 (24). Matrix metalloprotease 8 (MMP8) is a collagenase with pro-inflammatory effects in infection (25, 26). We have previously demonstrated that elevated MMP8 and interleukin-8 and low platelet counts were associated with a higher mortality risk from neonatal sepsis (27). Future research is necessary to determine whether these candidate genes –either alone or in combination with protein biomarkers– may be used for prognostic and predictive enrichment in neonatal sepsis (28).

Our study has several limitations. Our study had a limited number of subjects, highlighting the need to conduct studies enrolling neonates with sepsis to understand the disease pathophysiology better. Second, we do not yet have a consensus definition of neonatal sepsis (29), which introduces additional variability, potentially confounding the results. Furthermore, organ dysfunction definitions were not consistent across the datasets. Nonetheless, we investigated signatures associated with mortality – an unambiguous measure – as our primary outcome. However, the low numbers of non-survivors across datasets contribute to class imbalance, necessitating robust statistical approaches to adjust for confounders. Next, the datasets reflect gene expression at a single time point early in the disease. We could not assess for temporal changes that might reveal dynamic immune changes that occur with neonatal sepsis.

Furthermore, our study utilized computational bioinformatics to infer enriched pathways, transcriptional factors, and cell-type-specific signaling according to mortality or discovered endotypes without corroborative experiments to support our findings. These approaches rapidly evolve, and new methodologies could alter the results described here. As such, our study shows subtyping neonatal sepsis based on gene-expression profiles is feasible, and future efforts should bolster inclusion of neonates while narrowing the search for upstream mechanistic targets that require orthogonal validation and rigorous mechanistic testing. Lastly, the patient endotypes identified had prognostic implications. Yet, whether the endotypes demonstrate heterogeneous responses to NICU interventions, including immunomodulatory therapies such as corticosteroids, remains to be tested.

## 5 Conclusions

Neonatal sepsis is a major cause of morbidity and mortality, yet its biological heterogeneity limits targeted interventions. We identified distinct neonatal sepsis endotypes using gene-expression profiling, with endotype A associated with higher mortality and immune dysregulation. Our findings suggest that immune dysfunction, rather than traditional clinical factors, drives poor outcomes. These results highlight the potential of transcriptomic profiling for risk stratification and precision medicine approaches in neonatal sepsis.

## Data Availability

Publicly available datasets were analyzed in this study. This data can be found here: https://www.ncbi.nlm.nih.gov/gds datasets: GSE25504 and GSE69686</b>.
