# Supplementary material for: Transcriptomic mortality signature defines high-risk neonatal sepsis endotype
Source: Front Immunol. 2025 Jun 27;16:1601316. doi: 10.3389/fimmu.2025.1601316 (PMC12245816; doi:10.3389/fimmu.2025.1601316)
Supplement: Supplementary file 1 [file Table1.docx]

**Online Supplement to Transcriptomic Mortality Signature Defines High-Risk Neonatal Sepsis Endotype**

Faris N. Al Gharaibeh, Min Huang, James L. Wynn, Rishikesan Kamaleswaran, and Mihir R. Atreya.

**Contents:**

**Supplementary Tables:**

**Table S1. Differentially Expressed Genes According to Mortality…………………………...2**

**Table S2. Key Transcriptional Factors by Chip Enrichment Analysis………………………5**

**Table S3. Key Transcriptional Factors by Ingenuity Pathway Analysis……………………..9**

**Table S4. Clinical variables comparing non-survivors and survivors in Cluster A………………………………………………………………………………………….............10**

**Supplementary Figures:**

**Figure S1. Key Transcriptional Factors by Chip Enrichment Analysis…………….............11**

**Table S1. Differentially Expressed Genes According to Mortality**

| Gene | logFC | AveExpr | P.Value | adj.P.Val |
| --- | --- | --- | --- | --- |
| PRTN3 | 4.821247 | 8.547795 | 0.00106 | 0.049775 |
| BPI | 3.889558 | 8.387426 | 2.98E-06 | 0.000889 |
| MPO | 3.63577 | 9.167598 | 0.000933 | 0.045712 |
| MS4A3 | 3.144197 | 8.300925 | 1.22E-05 | 0.002104 |
| CTSG | 2.945614 | 7.128453 | 0.000272 | 0.019473 |
| MT1G | 2.805801 | 6.035782 | 0.00056 | 0.031077 |
| DEFA4 | 2.509028 | 8.584251 | 0.000966 | 0.046543 |
| RNASE2 | 2.379471 | 9.760973 | 3.55E-05 | 0.004393 |
| RNASE3 | 2.318368 | 7.336487 | 1.06E-05 | 0.00191 |
| CEBPE | 2.292118 | 7.04385 | 9.32E-06 | 0.00191 |
| SLPI | 2.257702 | 7.100813 | 0.000237 | 0.018138 |
| SERPINB10 | 2.165225 | 5.650483 | 0.001014 | 0.048029 |
| S100P | 1.958679 | 9.292439 | 0.000585 | 0.032138 |
| CD24 | 1.91805 | 9.190694 | 1.35E-06 | 0.000595 |
| NUCB2 | 1.885574 | 7.485612 | 1.37E-07 | 0.000235 |
| GGH | 1.864203 | 5.924028 | 6.42E-08 | 0.000142 |
| PRC1 | 1.787007 | 6.165654 | 6.00E-06 | 0.001504 |
| CEP55 | 1.774998 | 5.852922 | 2.95E-05 | 0.003874 |
| GPI | 1.658824 | 8.815939 | 0.000681 | 0.036163 |
| KIF11 | 1.634631 | 6.594276 | 1.02E-06 | 0.000584 |
| ATP8B4 | 1.626029 | 7.302033 | 6.50E-05 | 0.007104 |
| CCNB2 | 1.583582 | 6.042296 | 8.61E-06 | 0.001875 |
| PRSSL1 | 1.566984 | 6.040881 | 8.32E-06 | 0.001875 |
| TYMS | 1.550439 | 7.770582 | 1.12E-05 | 0.001962 |
| RRM2 | 1.521515 | 6.811001 | 5.20E-06 | 0.001368 |
| RAD51AP1 | 1.519957 | 4.950027 | 1.91E-07 | 0.000238 |
| SPC25 | 1.510382 | 4.991986 | 2.53E-06 | 0.000845 |
| CCNA2 | 1.50651 | 6.919644 | 8.66E-06 | 0.001875 |
| SERPINB2 | 1.497331 | 6.227215 | 0.000329 | 0.022019 |
| MT1X | 1.490178 | 6.970352 | 0.00036 | 0.023489 |
| ATAD2 | 1.472057 | 6.227464 | 3.19E-07 | 0.00032 |
| CCNE2 | 1.435531 | 5.606275 | 5.96E-07 | 0.000387 |
| MYB | 1.417636 | 7.028899 | 7.14E-06 | 0.001751 |
| MCM10 | 1.414794 | 5.2589 | 2.81E-08 | 7.76E-05 |
| ASPM | 1.393632 | 5.817374 | 2.69E-06 | 0.000871 |
| SLC44A1 | 1.390945 | 7.677925 | 1.79E-05 | 0.002748 |
| UBE2C | 1.367086 | 6.384706 | 0.000266 | 0.01942 |
| NUSAP1 | 1.35626 | 7.010639 | 4.79E-07 | 0.000353 |
| CENPF | 1.351666 | 5.720306 | 3.49E-07 | 0.000321 |
| MT1F | 1.335596 | 7.722193 | 0.001021 | 0.048174 |
| IGFBP7 | 1.335521 | 7.003187 | 4.73E-06 | 0.001273 |
| CENPE | 1.322429 | 5.854102 | 1.56E-06 | 0.000595 |
| C1QC | 1.312568 | 4.771434 | 0.000226 | 0.017557 |
| AIG1 | 1.288144 | 6.595883 | 0.000171 | 0.014447 |
| FUT4 | 1.276247 | 6.977735 | 0.000549 | 0.031077 |
| AURKA | 1.259062 | 6.86219 | 9.77E-06 | 0.00191 |
| ANLN | 1.256385 | 6.037354 | 3.89E-05 | 0.004662 |
| MKI67 | 1.254967 | 5.783315 | 9.85E-06 | 0.00191 |
| GINS2 | 1.254178 | 5.101058 | 9.80E-05 | 0.009409 |
| BUB1 | 1.252067 | 5.620002 | 3.56E-05 | 0.004393 |
| CENPN | 1.248004 | 5.683586 | 4.29E-07 | 0.000353 |
| TSPAN2 | 1.231775 | 7.61003 | 7.71E-05 | 0.00809 |
| FBXO5 | 1.219491 | 5.929786 | 2.38E-05 | 0.003374 |
| HMMR | 1.218019 | 5.837827 | 3.24E-05 | 0.004204 |
| NCAPG2 | 1.216528 | 5.969192 | 1.02E-08 | 3.74E-05 |
| CDKN3 | 1.215672 | 5.783433 | 2.53E-06 | 0.000845 |
| KIF15 | 1.214235 | 5.117292 | 1.50E-06 | 0.000595 |
| TTK | 1.212979 | 4.649868 | 8.67E-05 | 0.008776 |
| GSTM3 | 1.210338 | 4.502468 | 0.000384 | 0.024783 |
| PLK4 | 1.208772 | 4.761726 | 1.06E-09 | 1.17E-05 |
| DTWD2 | 1.198523 | 5.364025 | 0.000444 | 0.026737 |
| POLQ | 1.195771 | 4.820467 | 2.28E-09 | 1.26E-05 |
| KIF14 | 1.18772 | 5.768375 | 4.58E-05 | 0.005208 |
| RRAGD | 1.180615 | 7.11877 | 0.0008 | 0.040142 |
| TPX2 | 1.17793 | 6.205908 | 0.000261 | 0.019353 |
| MSRB3 | 1.175946 | 5.133758 | 1.16E-06 | 0.000584 |
| EAF2 | 1.17356 | 6.8657 | 1.44E-06 | 0.000595 |
| HSD17B12 | 1.163496 | 7.443579 | 2.57E-05 | 0.003596 |
| NCAPG | 1.161729 | 5.293771 | 1.54E-06 | 0.000595 |
| WBP5 | 1.139196 | 4.963077 | 0.000156 | 0.013539 |
| GMNN | 1.138704 | 5.978823 | 1.24E-06 | 0.000595 |
| KIFC1 | 1.138447 | 5.407022 | 1.02E-05 | 0.00191 |
| TUBB | 1.137353 | 8.849719 | 0.000119 | 0.010886 |
| CKAP2L | 1.129787 | 5.3372 | 4.25E-05 | 0.00505 |
| RRM1 | 1.124298 | 5.405263 | 0.000284 | 0.019738 |
| PSAT1 | 1.118791 | 4.795082 | 0.00027 | 0.019473 |
| C1orf135 | 1.111484 | 6.754775 | 6.79E-05 | 0.007276 |
| CDC20 | 1.095836 | 6.153812 | 9.04E-05 | 0.009072 |
| HJURP | 1.094419 | 5.962952 | 0.000185 | 0.015094 |
| GFI1 | 1.088919 | 6.378369 | 2.76E-06 | 0.000871 |
| SLC27A2 | 1.088554 | 4.698775 | 4.55E-05 | 0.005208 |
| APOBEC3B | 1.083714 | 5.713262 | 0.000354 | 0.02339 |
| LXN | 1.081393 | 6.347345 | 0.000257 | 0.019324 |
| ORC1L | 1.070145 | 5.188492 | 1.94E-07 | 0.000238 |
| KIF23 | 1.068641 | 5.289632 | 4.64E-06 | 0.001273 |
| KIAA1524 | 1.068477 | 5.095211 | 1.12E-06 | 0.000584 |
| NDC80 | 1.06831 | 5.920716 | 8.28E-06 | 0.001875 |
| MND1 | 1.056424 | 5.331391 | 9.98E-06 | 0.00191 |
| DTL | 1.05478 | 5.008506 | 1.58E-05 | 0.002521 |
| MCM4 | 1.053617 | 5.468533 | 0.000112 | 0.01043 |
| RAB13 | 1.051153 | 6.359438 | 2.27E-05 | 0.003258 |
| STIL | 1.012209 | 6.480596 | 9.32E-05 | 0.009189 |
| TRIP13 | 1.00975 | 5.067128 | 4.76E-07 | 0.000353 |
| PPP1R3C | 1.008978 | 4.433003 | 1.39E-05 | 0.002293 |
| H2AFZ | 1.008138 | 8.643212 | 0.000247 | 0.018825 |
| BARD1 | 1.003294 | 6.410839 | 1.07E-05 | 0.00191 |
| RALGDS | -1.00565 | 7.191678 | 4.44E-05 | 0.005198 |
| BCL11B | -1.22385 | 7.407941 | 0.000402 | 0.025644 |
| HLA-F | -1.34826 | 8.007633 | 0.000624 | 0.03361 |
| RARRES3 | -2.01738 | 7.298432 | 0.000604 | 0.032872 |

**Table S2. Key Transcriptional Factors by Chip Enrichment Analysis**

| Rank | TF | Score | Overlapping Genes |
| --- | --- | --- | --- |
| 1 | LTF | 0.0006143 | SERPINB10,ATP8B4,MS4A3,CRISP3,DEFA4,RNASE3,MMP8,MPO,LCN2,CHI3L1,CTSG,BPI,CEACAM8,CAMP |
| 2 | CEBPE | 0.0007123 | ATP8B4,MS4A3,RRM2,IL1R2,MMP8,MPO,SLPI,CLEC5A,MYB,LCN2,OLR1,CTSG,CD24 |
| 3 | E2F8 | 0.001229 | RAD51AP1,CCNB2,GINS2,ANLN,RRM2,TTK,MCM6,TYMS,CEP55 |
| 4 | ZNF215 | 0.001425 | GINS2,RRM2,TTK,TYMS,RAD51AP1,CCNB2,ANLN,MYB,MCM6,CEP55,METAP2,SPC25 |
| 5 | MYB | 0.001843 | VAMP8,ATP8B4,MS4A3,RNASE3,ERG,MCM6,TYMS,MPO |
| 6 | E2F7 | 0.001867 | RAD51AP1,CCNB2,GINS2,RRM2,TTK,MCM6,TYMS,CEP55,SPC25 |
| 7 | ZNF93 | 0.002137 | GINS2,RRM2,TTK,TYMS,RAD51AP1,CCNB2,ANLN,MYB,TIMP3,MCM6,CEP55,SPC25 |
| 8 | RFX8 | 0.002457 | SERPINB10,ATP8B4,MS4A3,CLEC5A,MYB,CTSG,RNASE3,MPO |
| 9 | CENPA | 0.002489 | RAD51AP1,CCNB2,GINS2,RRM2,TTK,MCM6,TYMS,CEP55,SPC25 |
| 10 | ZNF695 | 0.002849 | RAD51AP1,CCNB2,GINS2,ANLN,RRM2,MYB,TTK,MCM6,TYMS,CEP55,SPC25 |
| 11 | GFI1 | 0.003071 | ATP8B4,DEFA4,MYB,CTSG,BPI,RNASE3,MPO |
| 12 | MYBL2 | 0.003111 | RAD51AP1,CCNB2,GINS2,RRM2,TTK,MCM6,TYMS,CEP55,SPC25 |
| 13 | FOXM1 | 0.003367 | VAMP8,CCNB2,ANLN,RRM2,MYB,TTK,TYMS,CEP55 |
| 14 | ZNF519 | 0.003561 | RAD51AP1,CCNB2,GINS2,ANLN,RRM2,MYB,TTK,MCM6,TYMS,CEP55,SPC25 |
| 15 | ZNF124 | 0.003734 | RAD51AP1,CCNB2,GINS2,RRM2,TTK,MCM6,TYMS,CEP55,SPC25 |
| 16 | ZNF367 | 0.004274 | RAD51AP1,CCNB2,GINS2,ANLN,RRM2,MYB,TTK,MCM6,TYMS,CEP55,SPC25 |
| 17 | PRMT3 | 0.0043 | RAD51AP1,ANLN,RRM2,TTK,MCM6,CEP55 |
| 18 | AHRR | 0.004356 | RAD51AP1,CCNB2,GINS2,RRM2,TTK,MCM6,TYMS,CEP55,SPC25 |
| 19 | E2F1 | 0.004978 | RAD51AP1,CCNB2,GINS2,RRM2,TTK,MCM6,TYMS,CEP55,SPC25 |
| 20 | ZBED2 | 0.004986 | COL17A1,ANLN,RRM2,SLPI,TCN1,IL18,LCN2,OLR1,TTK,TYMS,CEP55 |
| 21 | MYBL1 | 0.005698 | RAD51AP1,CCNB2,GINS2,ANLN,RRM2,MYB,TTK,MCM6,TYMS,CEP55,SPC25 |
| 22 | HOXA9 | 0.006143 | VAMP8,ATP8B4,MYB,ERG,TYMS |
| 23 | ZNF492 | 0.006223 | RAD51AP1,CCNB2,GINS2,RRM2,TTK,MCM6,TYMS,CEP55,SPC25 |
| 24 | LYL1 | 0.006734 | MS4A3,CLEC5A,MYB,PRG2,CHI3L1,BPI,MPO |
| 25 | DNMT1 | 0.006845 | RAD51AP1,CCNB2,GINS2,RRM2,TTK,MCM6,TYMS,CEP55,SPC25 |
| 26 | NFYB | 0.007371 | RAD51AP1,CCNB2,GINS2,TTK,METAP2 |
| 27 | TFDP1 | 0.007467 | RAD51AP1,CCNB2,GINS2,RRM2,TTK,MCM6,TYMS,CEP55,SPC25 |
| 28 | ZIC2 | 0.007985 | RAD51AP1,CCNB2,GINS2,TTK,CD24 |
| 29 | E2F4 | 0.008475 | RAD51AP1,GINS2,RRM2,TTK,MCM6,TYMS |
| 30 | HMGA1 | 0.008712 | RAD51AP1,CCNB2,GINS2,RRM2,TTK,MCM6,TYMS,CEP55,SPC25 |
| 31 | E2F2 | 0.009259 | RAD51AP1,CCNB2,GINS2,ANLN,RRM2,MYB,TTK,MCM6,TYMS,CEP55,SPC25 |
| 32 | EHF | 0.009828 | COL17A1,SLPI,TACSTD2,LCN2,CD24 |
| 33 | ZFP69B | 0.009956 | RAD51AP1,GINS2,RRM2,MCM6,TYMS,CEP55,SPC25 |
| 34 | CBX2 | 0.009972 | RAD51AP1,CCNB2,GINS2,ANLN,RRM2,MYB,TTK,MCM6,TYMS,CEP55,SPC25 |

**Table S3. Key Transcriptional Factors by Ingenuity Pathway Analysis**

| Rank | TF | Predicted State | z-score | p-value |
| --- | --- | --- | --- | --- |
| 1 | TBX3 | Activated | 5.099 | 4.52E-32 |
| 2 | E2F4 |  |  | 4.03E-28 |
| 3 | TCF3 | Inhibited | -2.605 | 4.66E-23 |
| 4 | ZBTB17 |  |  | 1.07E-21 |
| 5 | CCND1 | Activated | 2.543 | 1.03E-18 |
| 6 | TP53 | Inhibited | -2.997 | 2.46E-17 |
| 7 | E2F3 | Activated | 3.138 | 2.82E-17 |
| 8 | FOXM1 | Activated | 3.658 | 3.59E-16 |
| 9 | MYOD1 | Activated | 3 | 1.68E-15 |
| 10 | E2F1 | Activated | 3.383 | 2.11E-15 |
| 11 | COPS5 | Activated | 3.357 | 1.85E-14 |
| 12 | RB1 | Inhibited | -3.27 | 9.04E-14 |
| 13 | CEBPB | Activated | 3.307 | 2.24E-12 |
| 14 | NUPR1 | Inhibited | -3.5 | 7.71E-12 |
| 15 | TCF4 |  |  | 7.74E-12 |
| 16 | FOXO3 |  |  | 1.31E-11 |
| 17 | TRPS1 | Inhibited | -2.828 | 1.45E-11 |
| 18 | E2F2 |  | 1.964 | 3.22E-11 |
| 19 | SMARCB1 | Inhibited | -2.227 | 5.19E-11 |
| 20 | MYBL2 | Activated | 2.157 | 8.52E-11 |
| 21 | TFEB | Activated | 3.729 | 5.25E-10 |
| 22 | TAL1 | Activated | 3.162 | 8.29E-10 |
| 23 | GLIS3 |  |  | 8.4E-09 |
| 24 | FOXO1 | Activated | 2.359 | 1.28E-08 |
| 25 | RBL1 | Inhibited | -2 | 1.61E-08 |
| 26 | TBX2 | Activated | 2.646 | 2.72E-08 |
| 27 | SPI1 |  | -1.196 | 4.68E-08 |
| 28 | MRTFB |  | -1.134 | 7.19E-08 |
| 29 | YAP1 | Activated | 2.407 | 7.64E-08 |
| 30 | YY1 |  |  | 1.11E-07 |
| 31 | GON4L |  |  | 1.71E-07 |
| 32 | MRTFA |  | -1.134 | 1.78E-07 |
| 33 | RRP1B |  |  | 1.88E-07 |
| 34 | TBX21 |  |  | 3.21E-07 |

**Table S4. Clinical variables comparing non-survivors and survivors in Cluster A**

^a^ Calculated using the Mann-Whitney test Calculated using the Fisher’s exact test

^b^ Calculated using the Fisher’s exact test

| Characteristic | Non-survivors n=5 (22%) | Survivors  n=18 (78%) | p-value |
| --- | --- | --- | --- |
| GA at Birth Weeks [IQR] | 27 [24-29] | 27 [25-30] | 0.79^a^ |
| Sex at Birth: Female  male | 1 (20%)  4 (80%) | 8 (44%)  10 (56%) | 0.61^b^ |
| Early vs. Late | 2 (40%) | 2 (11%) | 0.19^b^ |
| Pathogen Identified | 5 (100%) | 12 (67%) | 0.28^b^ |
| Total White Count /mm^3^ [IQR] | 9900 [1700-15000] | 11700 [6450-23250] | 0.22^a^ |
| Respiratory Dysfunction | 5 (100%) | 13 (72%) | 0.55 ^b^ |
| Cardiac Dysfunction | 5 (100%) | 9 (50%) | 0.12 ^b^ |


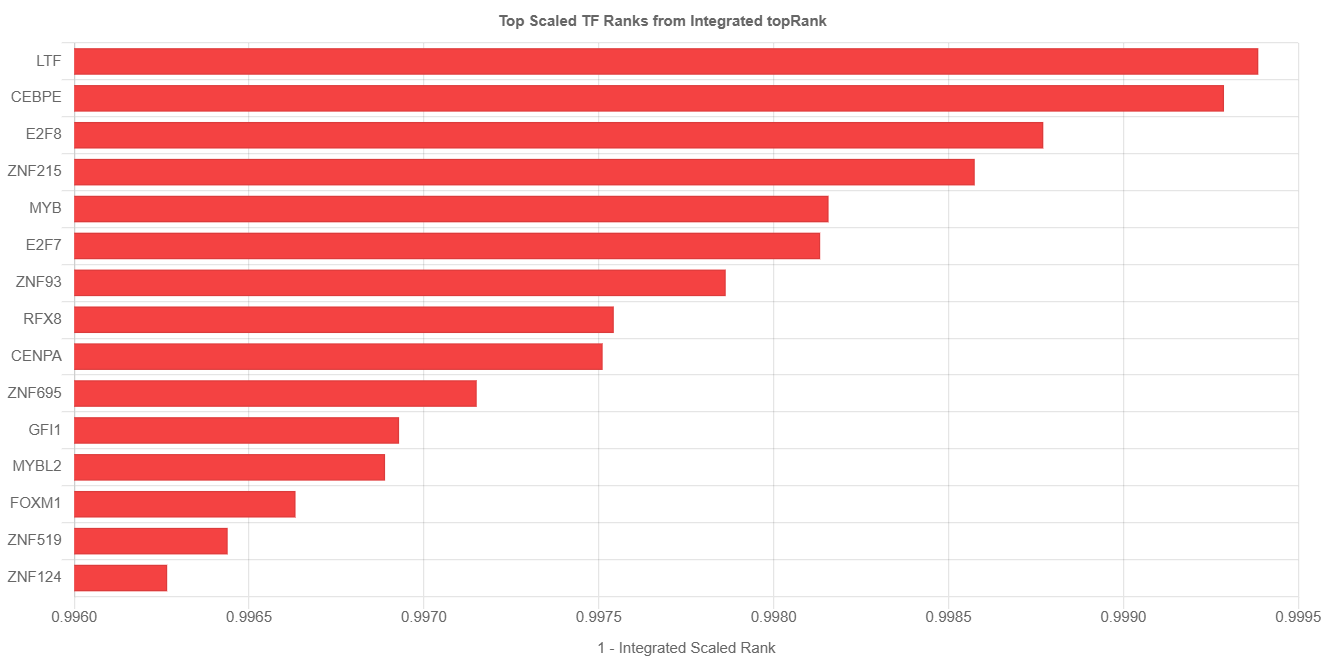


**Figure S1. Key Transcriptional Factors by Chip Enrichment Analysis.** The top-scaled transcriptional factors are shown on the y-axis.
